# Managing Complexity in Rett Syndrome with a Focus on Respiratory Involvement: A Tertiary Center Experience

**DOI:** 10.3390/children12091181

**Published:** 2025-09-04

**Authors:** Adele Corcione, Luigi Antonio Del Giudice, Simona Basilicata, Mariantonia Maglio, Salvatore Aiello, Raffaele Cerchione, Anna Annunziata, Alessandro Amaddeo, Melissa Borrelli

**Affiliations:** 1 Department of Translational Medical Sciences, Federico II University, 80131 Naples, Italy; adele.corcione@unina.it (A.C.); luigiantonio.delgiudice@unina.it (L.A.D.G.); simona.basilicata@unina.it (S.B.); raffaele.cerchione@unina.it (R.C.); melissa.borrelli@unina.it (M.B.); 2 European Laboratory for the Investigation of Food-Induced Diseases, Federico II University, 80131 Naples, Italy; 3 Department of Translational Medical Sciences, Child and Adolescents Neuropsychiatry, Federico II University, 80131 Naples, Italy; salvatore.aiello2@unina.it; 4 Respiratory Physiopathology and Rehabilitation Unit, AORN dei Colli, 80131 Naples, Italy; anna.annunziata@gmail.com; 5 Emergency Department, IRCCS Burlo Garofolo, 34137 Trieste, Italy; alessandro.amaddeo@burlo.trieste.it

**Keywords:** Rett syndrome, comorbidities, respiratory infections, sleep-disordered breathing, apnea

## Abstract

Background: Rett syndrome (RS) is a rare neurodevelopmental disorder primarily affecting females, characterized by severe neurological impairment and complex comorbidities, including epilepsy, scoliosis, and respiratory dysfunction. Respiratory complications, such as recurrent infections and sleep-disordered breathing (SDB), are increasingly recognized as significant contributors to morbidity. This study aimed to evaluate the prevalence, severity, and management of major comorbidities—including epilepsy, scoliosis, respiratory infections, and SDB—in a pediatric cohort with genetically confirmed RS. Methods: We conducted a retrospective review of medical records from 23 female patients under 18 years of age with MECP2 mutations, referred to our tertiary care center from 2021 to 2025. Data on epilepsy, scoliosis, respiratory infections, and nutritional status were collected. The presence of SDB was assessed through overnight home polygraphy (oPG) and transcutaneous carbon dioxide monitoring in selected cases. Results: Epilepsy affected 65% of patients, all with good seizure control. Scoliosis was present in 52%, with two patients requiring spinal surgery. At least one episode of lower respiratory tract infection (LRTI) was presented in 39% of our girls. LRTIs positively correlated with the number of hospitalization and antibiotic treatment. Among patients undergoing oPG, 67% presented obstructive sleep apnea, with its severity positively correlating with the frequency of lower respiratory infections. Severe nocturnal hypercapnia was documented in three patients, leading to non-invasive or invasive ventilation. Conclusions: Our findings highlight the high prevalence of sleep-related respiratory disorders and their association with respiratory infections in children with RS. Systematic respiratory assessment, including sleep studies, and early implementation of airway clearance techniques and ventilatory support are crucial to improving clinical outcomes in this vulnerable population.

## 1. Introduction

Rett syndrome (RS) is a rare neurodevelopmental disorder primarily affecting females, caused by de novo mutations in the methyl-CoPG-binding protein 2 (MECP2) gene located on the X chromosome [1]. Atypical forms of RS may be caused by mutations in cyclin-dependent kinase-like 5 (CDKL5) [2]. RS is estimated to affect approximately 1 in 10,000 to 15,000 female births worldwide, making it one of the most common causes of intellectual disability in females [3]. The clinical presentation of classic RS is complex and evolves over time; initially, affected individuals experience a period of seemingly normal development for the first 6–18 months of life, this is followed by a rapid regression phase, during which previously acquired skills, such as purposeful hand movements and spoken language, are lost. Subsequently, a period of stabilization usually occurs, followed by further motor deterioration. Loss of intentional hand movements; the emergence of stereotypical hand movements, such hand washing; vocal language loss; aberrant gait; and stunted growth are some of the main clinical characteristics of RS. This condition is associated with relevant medical comorbidities, including seizures, scoliosis, gastrointestinal issues, and breathing abnormalities. Affected people may differ widely in the presentation and intensity of these symptoms, which makes diagnosis and treatment more difficult [4]. There is currently no cure for RS, and its management focuses on symptomatic treatment and supportive care. A multidisciplinary approach involving neurologists, pulmonologists, physiotherapists, speech therapists, and other specialists is essential to address the different needs of the patients [4,5]. They may require variable therapeutic interventions ranging from anticonvulsant medications to physical therapy, nutritional support, and respiratory management [6].

The aim of our study was to analyze the prevalence, severity, and therapeutic management of major comorbidities such as scoliosis, epilepsy, and respiratory infections in a cohort of patients with RS. Moreover, the prevalence and pattern of sleep-related respiratory disorders, as well as their relationship with other comorbidities, were evaluated in our study.

## 2. Materials and Methods

We retrospectively collected data from female patients with a confirmed diagnosis of RS with MECP2 gene mutation and age <18 years, referred to our academic tertiary care department, Federico II University, Naples, Italy, from April 2021 to April 2025.

A diagnostic and therapeutic care plan is active for patients affected by RS at our department; the management of the patients involves an interdisciplinary team approach, consisting of pediatric pulmonologists, physiatrists, orthopedists, respiratory physiotherapists, gastroenterologists, and neuropsychiatrists. We collected patients’ medical records showing data including age at evaluation, epilepsy, respiratory infections, scoliosis, hospitalizations, therapeutic strategies, nutrition feeding modalities, and sleep-related breathing disorders. Concerning respiratory infections (upper respiratory tract infections, URTIs; lower respiratory tract infections, LRTIs), hospitalizations, and antibiotic treatments, we evaluated events occurring in the previous year. Data from patients were anonymously recorded using a standardized database.

All patients provided written informed consent for the publication of their anonymized clinical and laboratory data.

### 2.1. Sleep Study by Polygraphy

Data about sleep-related breathing disorder were obtained by an oPG, using a cardiorespiratory device (NoxT3™—PSG System manufactured by NOX Medical, Reykjavík, Iceland).

The sleep study was performed under clinical wellbeing conditions, clinically controlled epilepsy, and absence of respiratory flare-ups. The parents/caregivers of the patients were trained to set up the equipment.

oPG measurements included: nasal airflow (nasal pressure transducer), oxygen saturation (SpO_2_) and cardiac rate (pulse oximetry), thoracic and abdominal movements (inductance plethysmography), and body position.

The recordings were manually scored by two pediatricians of the team with expertise in sleep medicine, according to American Academy of Sleep Medicine guidelines [7].

The following cardiorespiratory parameters were evaluated:

- Total sleep time (TST): the total recording time (in minutes) excluding periods of artifacts and gross body movements.
- Snoring time (%): Percentage of the TST spent snoring.
- Apnea–hypopnea index (AHI): The number of apneas and hypopneas per hour of sleep.
- Obstructive apnea–hypopnea index (oAHI): The number of obstructive and mixed apneas/hypopneas per hour of sleep.
- Central apnea–hypopnea index (CAHI): The number of central apneas/hypopneas per hour of sleep.
- Oxygen desaturation index (ODI): The number of ≥ 3% SpO_2_ desaturation events per hour of sleep.
- Mean SpO_2_.
- Percentage of the TST spent with an SpO_2_ < 90% (T90).

Obstructive sleep apnea (OSA) was defined based on an oAHI higher than 1 event/hour. The severity of OSA was graded, based on oAHI, as follows: mild = 1 < oAHI ≤ 5 events/h; moderate = 5 < oAHI < 10 events/h; severe = oAHI ≥ 10 events/h.

As regards central sleep apnea (CSA), a CAHI ≤ 1 event/h was considered normal, whereas a value of CAHI ≥ 5 event/h was considered clinically significant [8].

Patients with severe sleep apnea performed transcutaneous carbon dioxide (PtcCO_2_) recording using the SenTec Digital Monitor (SenTecInc, Therwil, Switzerland) during an overnight hospital stay.

Mean and maximum PtcCO_2_ and the percentage of TST spent with a PtcCO_2_ > 50 mmHg were reordered. Nocturnal hypoventilation was defined as a PtcCO_2_ > 50 mmHg for > 25% of TST [7].

### 2.2. Statistical Analysis

The variables studied had a normal distribution. Comparison between groups was performed using unpaired *t*-test. Mann–Whitney test was used to analyze non-parametric variables. To correlate variables, we employed the Pearson test. To compare the percentage of positivity, we used the χ^2^ test. Means and standard deviations (SDs) were reported for continuous variables; median and range were reported for non-parametric variables, and frequencies and percentages were reported for categorical variables. A *p* value < 0.05 was considered statistically significant. We employed GraphPad Prism 2006 Software to assess statistics.

## 3. Results

### 3.1. Clinical Data

Twenty-three girls with RS, mean age at enrolment 9.2 ± 4.2 years old, were included in the study. Their clinical features and therapeutic management are summarized in Table 1 and Table 2.

Concerning comorbidities, epilepsy, managed with antiepileptic drugs, was observed in 15/23 (65%) patients.

Additionally, one patient (n. 22) took antiepileptic therapy due to the presence of abundant multifocal interictal epileptiform abnormalities on electroencephalography, without symptomatic epilepsy. All of them had good control of seizures.

Scoliosis, another common comorbidity, was present in 12/23 (52%) patients. One of them (n. 19) underwent arthrodesis surgery at the age of 11 years. Furthermore, 12 out of 23 (52%) patients were wheelchair dependent; 7/12 (58%) had scoliosis, and 5/12 (42%) did not have it. Fisher test analysis did not show statistical significance between the two percentages.

**Figure 1:**
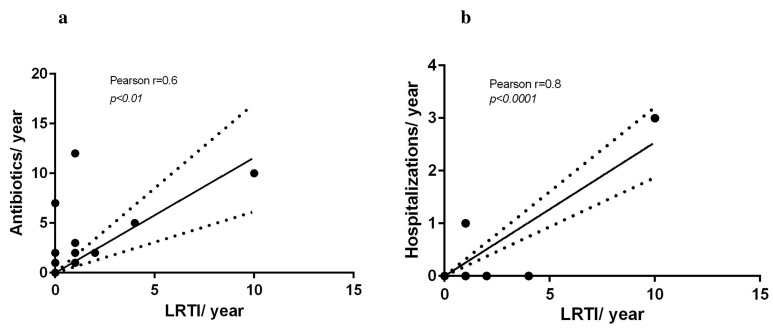
(**a**,**b**) Pearson correlation analysis. In the RS patients, the number of lower respiratory tract infections (LRTIs) positively correlated with both the number of antibiotic treatments, r = 0.6, *p* < 0.01 (**a**), and the number of hospitalizations, r = 0.8, *p* < 0.001 (**b**), during the previous year.

Investigating respiratory issues, 13/23 (56%) patients presented at least one episode of URTI, and 9/23 (39%) presented at least one episode of LRTI. The URTI number (median = 1; range 0–12) did not differ from the LRTI one (0; 0–10). The median value of antibiotic administration was 1 (range 0–12). LRTIs, but not URTIs, showed a strong positive correlation with antibiotic therapy (Pearson r = 0.6, *p* < 0.005). Furthermore, the LRTI number positively correlated with the number of hospitalizations due to respiratory issues (Pearson r = 0.8, *p* < 0.0001) (Figure 1a, b); this correlation was not found for the URTI number.

The number of LRTIs and URTIs was not statistically different in the presence of comorbidities such as epilepsy or scoliosis.

Four patients (17% n. 9, 15, 21, 22) underwent an airway clearance technique (ACT) with cough assistance.

Two patients (n. 19, 22) underwent an intensified ACT program with cough assistance to prevent respiratory infections during the post-operation period. One of these patients underwent arthrodesis surgery for severe scoliosis; the other one had a laparoscopic left salpingo-ovariectomy for an immature cystic teratoma. Both required invasive ventilation through endotracheal intubation (ET-IV) for about 3 h. Successively, admitted to an ordinary pediatric ward, they received non-invasive ventilatory support using high-flow nasal cannula oxygen therapy (HFNC) for 24 h and ACT by mechanical cough assistance three times/day for seven days. They did not present any post-operative complications.

### 3.2. Polygraphy Data

Fifteen patients (65%), mean age 9.8 ± 4.4 years old, underwent oPG as they presented one or more of the following symptoms: snoring, obstructive apnea, hypotonia, and scoliosis. All data of oPG are summarized in Table 3.

Median oAHI was 3.7 events/h (range 0–11.1); median CAHI was 0.4 events/h (0–7.2). No correlation was found between oAHI or CAHI and age of girls.

The median ODI was 3.8 (range 0.4–37); the median value of mean SpO_2_ and T90 were 96 (range 92–97) and 0.6 (0–2.9), respectively.

**Figure 2:**
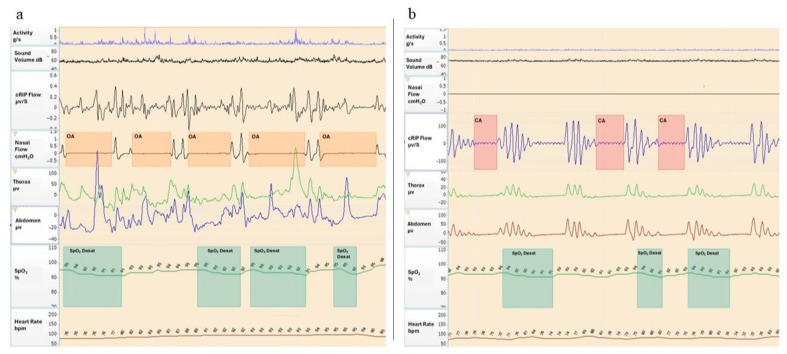
(**a**,**b**) Polygraphic recordings from two patients with RS. Cardiorespiratory monitoring assessed the following parameters: activity (g/s); sound volume (dB); nasal flow (cmH_2_O); cRIP flow, i.e., respiratory inductance plethysmography flow (µv/S); thorax (µv) and abdomen (µv), i.e., chest and abdominal movements; SpO_2_, i.e., oxygen saturation (%); and heart rate (bpm). (**a**) The monitoring shows several events of obstructive apnea (OA) characterized by a deep fall in the nasal pressure ≥ 90%, increase in respiratory effort with chest and abdominal movements, and oxygen desaturation (SpO_2_ Desat) > 3%. (**b**) The tracing shows three events of central apnea (CA) characterized by airflow drop ≥ 90% and absence of respiratory effort, associated with oxygen desaturation (SpO_2_ Desat) > 3%.

According to oAHI, 10/15 (67%) patients had OSA: 6 of them (60%) mild OSA, 2 (20%) moderate OSA, and 2 (20%) severe OSA. One patient (n. 22) had clinically significant CSA (CAHI ≥ 5 events/h) (Figure 2a,b).

**Figure 3:**
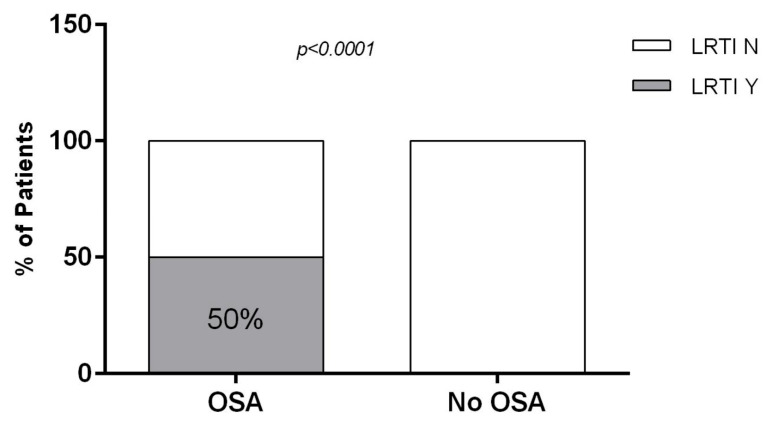
Chi square (χ^2^) test analysis. Comparison between the percentage of patients with (OSA) and without obstructive sleep apnea (No OSA) showing at least one lower respiratory tract infection (LRTI) over the previous year. The gray box indicates % of patients with ≥1 LRTI (LRTI Y); the white box represents % of patients with no LRTIs (LRTI N). The percentage of patients who experienced ≥1 LRTI was higher in the OSA group (50%) than in the No OSA group (0%), *p* < 0.0001.

Comparing the oPG data to respiratory problems, we showed that 50% of patients with OSA presented at least one LRTI in comparison to the group without OSA who did not have LRTIs (*p* < 0.0001) (Figure 3).

**Figure 4:**
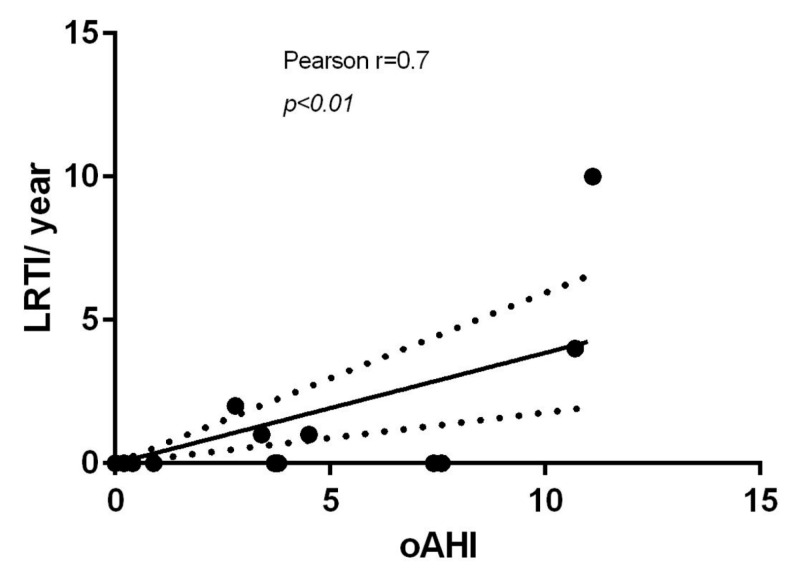
Pearson correlation analysis. The obstructive apnea–hypopnea index (oAHI) positively correlated (r = 0.70; *p* < 0.01) with the number of lower respiratory tract infections (LRTIs) over the previous year. The data indicated that a greater severity of obstructive sleep apnea is associated with a higher frequency of respiratory infections.

Moreover, oAHI positively correlated with episodes of LRTIs (Pearson r = 0.7, *p* < 0.01) (Figure 4) but not with URTIs.

In the case of comorbidities such as epilepsy and scoliosis, we found that the same percentage of patients had scoliosis and/or epilepsy, independent from the presence of OSA.

Three patients with severe apnea syndrome (patients n. 15, 21 OSA; patient n. 22 CSA; Table 3) underwent PtcCO_2_ recording. Mean values of mean and maximum PtcCO_2_ were 54.8 ± 2.16 mmHg and 60.86 ± 2.52 mmHg, respectively. They showed nocturnal hypercapnia, with a median value of % of TST with PtcCO_2_ > 50 mmHg of 91 (range 91–100). For this reason, patients 21 and 22 received non-invasive ventilatory support, and patient 15 underwent invasive ventilation via tracheostomy.

## 4. Discussion

Patients with RS present a complex and multifaceted clinical profile, with comorbidities affecting virtually every organ system [5]. The most frequently observed complications are neurological manifestations such as epilepsy and movement disorders; orthopedic problems, including scoliosis; and gastrointestinal and nutritional issues [9]. In our retrospective study, we first described the prevalence and therapeutic management of major comorbidities in a cohort of children and adolescents with RS. We found a high prevalence of epilepsy with more than two-thirds (70%) of our girls taking antiepileptic medications at the time of enrolment, as described in adults [9]. Our data also confirmed that scoliosis, which occurred in about half of our patients, is a common comorbidity, although with a lower prevalence, especially as regards corrective surgery, than that in a previous paper [9]. This could be explained by the average age of our population (mean age at enrolment 9.2 ± 4.2 years old); in fact, it is described that 75% of RS patients develop scoliosis by the age of 15 years and that severe conditions requiring surgery are rare before age 9 years [10]. So, in conclusion, both epilepsy and scoliosis are early-onset comorbidities that should be recognized and treated appropriately. Indeed, early therapeutic interventions have shown improvement in survival as well as in the control of other comorbidities, such as respiratory infections [10]. About nutritional issues, almost all the patients of our pediatric population received food orally (solid or semi-solid feeding), showing a good weigh gain and no reported symptoms and signs of aspiration. Only 9% had enteral nutrition exclusively through a PEG for documented risk of aspiration. The risk of inhalation seems to increase with age; in fact, the percentage of patients in our study with a PEG is lower than that of adults (27%) [9].

Our data showed that respiratory involvement, particularly respiratory infection, was an important condition with significant consequences such as use of antibiotic therapy and hospitalization. These data are consistent with the results of a recent study that underlines the risk of severe healthcare-associated infections, commonly sustained by antimicrobial-resistant microorganisms, in patients with complex and chronic conditions like RS [5]. Moreover, it is known that the hospitalization of children with complex disease affects the quality of life of patients and their families, due to the detachment from their place of origin, economic issues, and work difficulties for parents. In addition, the management of these patients requires highly specialized healthcare structures and several subspecialists. However, these centers are not always near the patients’ residences, due to the non-homogeneous geographical distribution of healthcare resources, worsening the discomfort of hospitalizations [11].

Although this topic is not addressed by currently available guidelines [12], an early daily physiotherapy program could be useful to prevent respiratory infections. As in other complex chronic conditions, the use of airway clearance techniques, such as mechanical cough assistance, could be useful for RS patients; therefore, we recommend intensifying the ACT program in case of a respiratory flare-up or during the post-operation period.

Regarding respiratory involvement, SDB is another important issue. It may be easily overlooked due to the clinical heterogeneity of RS and the high burden of other comorbidities that often dominate the clinical picture. However, the potential consequences of unrecognized SDB, including poor sleep quality, daytime somnolence, impaired cognitive functioning, and even cardiopulmonary complications, underscore the importance of systematically screening for these disturbances. In our study we performed oPG in 65% of the RS population; we recognize that this is a limitation. However, it is important to consider that the sleep study is poorly tolerated by patients and their caregivers, especially in the absence of symptoms. Finally, this method is expensive and time consuming. According to the recent Italian experts’ consensus [5], patients in our cohort presenting at least one symptom, such as snoring, obstructive apnea, hypotonia, and scoliosis, underwent sleep study. Although polysomnography (PSG) is the gold standard for the diagnosis of sleep-disordered breathing, it is an expensive, poorly tolerated, and time-consuming tool. Polygraphy is more affordable than PSG, showing good sensitivity and specificity in the presence of moderate–severe sleep apnea. In order to reduce the burden of hospitalizations, our patients underwent home oPG. This tool provides several benefits for the diagnosis of OSA, especially for pediatric populations, such as increasing diagnostic accuracy and simplifying the diagnostic process, with no significant difference in results compared with PSG [13].

Most of our patients (67%) had a diagnosis of OSA—moderate to severe in almost half of them. Central sleep apnea was found in only one patient (7%) with mild OSA. These data confirm the higher prevalence of obstructive respiratory events than central events in patients with RS [14,15].

Interestingly, no correlation was found between oAHI or CAHI and age, suggesting that severity may be independent of age. Regarding comorbidities, the presence of sleep apnea was not related to epilepsy, consistent with previous reports [15], and scoliosis.

However, our three patients with hypoventilation (n. 15, 21, 22) had scoliosis, in agreement with Hagebeuk et al. [16]. These data suggest that patients with RS and scoliosis should be carefully evaluated, in particular before surgery, by means of a polygraphy study along with non-invasive carbon dioxide monitoring [17].

Although the increased vulnerability to lower respiratory infection in patients with RS is known, and its relationship with age, mutation type, feeding, and walking status has already been investigated [18], to the best of our knowledge, our study for the first time evaluated the relationship between respiratory infections and sleep apnea. A significant positive correlation between OSA and LRTIs was observed, indicating that a greater severity of obstructive sleep apnea is associated with a higher frequency of respiratory infections. This finding could be explained by the potential role of sleep apnea in worsening airway inflammation and the mismatch in ventilation and perfusion, already known in subjects with RS [19].

Given the potential impact of SDB on overall health, we strongly support the inclusion of sleep-focused respiratory assessment in the routine clinical evaluation of patients with RS. While full polysomnography remains the gold standard, simplified tools such as oPG represent a feasible and informative alternative, particularly in fragile and uncooperative patients.

Notably, in our sample, respiratory events were often not associated with significant oxygen desaturation, underscoring the role of CO_2_ monitoring in detecting subtle hypoventilation that might otherwise go unnoticed. It would therefore be desirable to perform a sleep study using transcutaneous CO_2_ recording in addition to polygraphy. This is a limitation of our study due to the unavailability of home transcutaneous CO_2_ equipment, which was therefore reserved for the most severe cases.

Our findings highlight the high prevalence of sleep-related respiratory disorders and their association with respiratory infections in children with RS. Multidisciplinary services and programs are necessary to optimize health and wellbeing in this vulnerable population with enhancement of systematic respiratory assessments, including sleep studies, and implementation of early airway clearance techniques and ventilatory support.

## Figures and Tables

**Table 1:** Clinical features and therapeutic management of twenty-three girls with RS.

CLINICAL FEATURES	
Epilepsy [n. (%)]	15/23 (65)
Scoliosis [n. (%)]	12/23 (52)
Oral solid feeding [n. (%)]	16/23(70)
Oral semi-solid feeding [n. (%)]	5/23(21)
URTIs [n. (%)]	13/23 (56)
URTIs/last year [median (range)]	1 (0–12)
LRTIs [n. (%)]	9/23 (39)
LRTIs/last year [median (range)]	0 (0–10)
n. hospitalizations due to respiratory issues/last year [median (range)]	0 (0–3)
n. hospitalizations for non-respiratory-related issues/last year [median(range)]	0 (0–1)
**THERAPEUTIC MANAGEMENT**	
Antiepileptic drugs [n. (%)]	16/23 * (70)
Arthrodesis [n. (%)]	2/12 (16)
PEG [n. (%)]	2/23 (9)
Antibiotic treatment/last year [median (range)]	1 (0–12)
ACT [n. (%)]	4/23 (17)
Nocturnal ventilation [n. (%)]	3/23 (13)
Tracheostomy [n. (%)]	1/3 (1)

*Abbreviations:* RS, Rett syndrome; URTIs, upper respiratory tract infections; LRTIs, lower respiratory tract infections; PEG, percutaneous endoscopic gastrostomy; ACT, airway clearance technique. * A patient takes antiepileptic treatment based on electroencephalography abnormalities, in the absence of symptomatic epilepsy.

**Table 2:** Demographic and clinical data.

Patient	Age (Years)	Epilepsy	Scoliosis	PEG	URTIs (n/Year)	LRTIs (n/Year)	Antibiotics (n/Year)	H (n/Year)	ACT	Nocturnal Ventilation
1	16	N	Y	N	0	0	0	0	N	N
2	2.6	N	N	N	4	1	3	1	N	N
3	16.8	N	Y	N	0	0	1	0	N	N
4	5.4	Y	N	N	0	0	1	0	N	N
5	5.4	Y	N	N	3	0	2	0	N	N
6	5	N	N	N	10	0	0	0	N	N
7	11.8	Y	Y	N	0	0	1	0	N	N
8	8.2	N	Y	N	2	1	1	0	N	N
9	16	Y	Y	Y	0	1	2	0	Y	N
10	6.9	Y	N	N	0	0	1	0	N	N
11	7.5	Y	Y	N	2	0	2	0	N	N
12	7.6	Y	N	N	4	1	12	0	N	N
13	5.9	N	N	N	1	0	1	0	N	N
14	15.9	Y	Y	N	0	0	0	0	N	N
15	6.9	Y	N	Y	4	4	5	0	Y	Tracheostomy
16	12.5	Y	Y	N	1	1	2	0	N	N
17	9.6	Y	Y	N	0	0	7	0	N	N
18	13.7	Y	Y	N	1	0	1	0	N	N
19	13.5	Y	Y	N	0	0	1	0	N	N
20	6.5	Y	N	N	2	1	2	1	N	N
21	7.6	Y	N	N	0	10	10	3	Y	NIV
22	5.6	Y	Y	N	12	2	2	0	Y	NIV
23	10.1	N	N	N	4	0	0	0	N	N

*Abbreviations:* PEG, percutaneous endoscopic gastrostomy; URTIs, upper respiratory tract infections; LRTIs, lower respiratory tract infections; H, hospitalization for respiratory issues; ACT, airway clearance technique; n/year, number of events in the previous year; Y, yes; N, no; NIV, non-invasive ventilation.

**Table 3:** Polygraphic data.

	Patient	TST (Minutes)	Snoring Time (%)	AHI (Events/h)	oAHI (Events/h)	CAHI (Events/h)	ODI (Events/h)	Mean SpO_2_ (%)	T90 (%)	PtcCO_2_ > 50 mmHg (%)
**NO OSA** **oAHI < 1**	23	240	10.9	0.5	0	0	4.6	97	1.5	-
	17	297	1.6	0.8	0.2	0.6	1.2	97	0.1	-
	14	423	4.1	1.4	0.4	0.1	1.3	97	0.2	-
	18	449	9.5	0.4	0.4	0	0.5	97	0	-
	10	336	9.7	1.4	0.9	0.4	1.1	96	0	-
**MILD** **OSA** **1 < oAHI ≤ 5**	22	480	N/A	10.2	2.8	7.2	37	95	N/A	91
	16	335	7.3	5.9	3.4	2.5	0.4	95	1.8	-
	11	467	50	5.1	3.7	1.3	8.7	94	0.6	-
	5	382	19.7	4.7	3.8	0.9	3.5	97	0.2	-
	19	240	3.1	6.8	3.8	2.7	9.2	95	0.1	-
	9	394	0.3	4.5	4.5	0	3.1	96	1.4	-
**MODERATE** **OSA** **5 < oAHI < 10**	4	291	N/A	7.4	7.4	0	26.6	92	2.9	-
	3	466	11.1	8.2	7.6	0.6	4.2	96	1.9	-
**SEVERE OSA** **oAHI≥ 10**	15	240	N/A	11.5	10.7	0.4	18	95	N/A	100
	21	285	28.1	15.8	11.1	3.4	10.3	94	1.3	91
Mean ± DS		355 ± 88.2								
Median; range			9.6; 0.3–50	4.9; 0.4–15.8	3.7; 0–11.1	0.4; 0–7.2	3.8; 0.4–37	96; 92–97	0.6; 0–2.9	91; 91–100

*Abbreviations:* TST, total sleep time; snoring time (%): percentage of the TST spent snoring; AHI, apnea–hypopnea index; oAHI, obstructive apnea–hypopnea index; CAHI, central apnea–hypopnea index; ODI, oxygen desaturation index; SpO_2_, oxygen saturation by pulse oximetry; T90 (%), percentage of the TST spent with an SpO_2_ < 90%; tcPCO_2_ > 50 mmHg (%): the percentage of TST spent with a PtcCO_2_ > 50 mmHg; PtcCO_2_**,** transcutaneous carbon dioxide; N/A, not available.

## Data Availability

Data is contained within the article.

## Abbreviations

The following abbreviations are used in this manuscript:

RS	Rett syndrome
SDB	sleep-disordered breathing
oPG	overnight home polygraphy
LRTIs	lower respiratory tract infections
MECP2	methyl-CoPG-binding protein 2
CDKL5	cyclin-dependent kinase-like 5
URTIs	upper respiratory tract infections
TST	total sleep time
AHI	apnea–hypopnea index
oAHI	obstructive apnea–hypopnea index
CAHI	central apnea–hypopnea index
ODI	oxygen desaturation index
T90	percentage of the TST spent with an SpO_2_ < 90%
OSA	obstructive sleep apnea
CSA	central sleep apnea
PtcCO_2_	transcutaneous carbon dioxide
SD	standard deviation
ACT	airway clearance technique
ET-IV	endotracheal intubation
HFNC	high-flow nasal cannula oxygen therapy
PEG	percutaneous endoscopic gastrostomy
PSG	polysomnography

## References

[1] Amir R.E., Van den Veyver I.B., Wan M., Tran C.Q., Francke U., Zoghbi H.Y. (1999). Rett syndrome is caused by mutations in X-linked MECP2, encoding methyl-CpG-binding protein 2. Nat. Genet..

[2] Asuncion R.M.D., Ramani P.K. (2025). Rett Syndrome. StatPearls.

[3] Vilvarajan S., McDonald M., Douglas L., Newham J., Kirkland R., Tzannes G., Tay D., Christodoulou J., Thompson S., Ellaway C. (2023). Multidisciplinary Management of Rett Syndrome: Twenty Years’ Experience. Genes.

[4] Guan R., Li Q., Fu S., Writing Group For Practice Guidelines For Diagnosis And Treatment Of Genetic Diseases Medical Genetics Branch Of Chinese Medical Association (2020). Clinical practice guidelines for Rett syndrome. Chin. J. Med. Genet..

[5] Cherchi C., Chiappini E., Amaddeo A., Chiarini Testa M.B., Banfi P., Veneselli E., Cutrera R., panel for the Problems in Patients with Rett Syndrome (2024). Management of respiratory issues in patients with Rett syndrome: Italian experts’ consensus using a Delphi approach. Pediatr Pulmonol.

[6] Krajnc N. (2014). Severe respiratory dysrhythmia in Rett syndrome treated with topiramate. J. Child Neurol..

[7] Berry R.B., Budhiraja R., Gottlieb D.J., Gozal D., Iber C., Kapur V.K., Marcus C.L., Mehra R., Parthasarathy S., Quan S.F. (2012). Rules for scoring respiratory events in sleep: Update of the 2007 AASM Manual for the Scoring of Sleep and Associated Events. Deliberations of the Sleep Apnea Definitions Task Force of the American Academy of Sleep Medicine. J. Clin. Sleep Med..

[8] McLaren A.T., Bin-Hasan S., Narang I. (2019). Diagnosis, management and pathophysiology of central sleep apnea in children. Paediatr. Respir. Rev..

[9] Anderson A., Wong K., Jacoby P., Downs J., Leonard H. (2014). Twenty years of surveillance in Rett syndrome: What does this tell us?. Orphanet J. Rare Dis..

[10] Downs J., Torode I., Wong K., Ellaway C., Elliott E.J., Izatt M.T., Askin G.N., Mcphee B.I., Cundy P., Leonard H. (2016). Surgical fusion of early onset severe scoliosis increases survival in Rett syndrome: A cohort study. Dev. Med. Child Neurol..

[11] De Curtis M., Bortolan F., Diliberto D., Villani L. (2021). Pediatric interregional healthcare mobility in Italy. Ital. J. Pediatr..

[12] Fu C., Armstrong D., Marsh E., Lieberman D., Motil K., Witt R., Standridge S., Nues P., Lane J., Dinkel T. (2020). Consensus guidelines on managing Rett syndrome across the lifespan. BMJ Paediatr. Open.

[13] Borrelli M., Corcione A., Cimbalo C., Annunziata A., Basilicata S., Fiorentino G., Santamaria F. (2023). Diagnosis of Paediatric Obstructive Sleep-Disordered Breathing beyond Polysomnography. Children.

[14] Sarber K.M., Howard J.J.M., Dye T.J., Pascoe J.E., Simakajornboon N. (2019). Sleep-Disordered Breathing in Pediatric Patients With Rett Syndrome. J. Clin. Sleep Med..

[15] Amaddeo A., De Sanctis L., Arroyo J.O., Khirani S., Bahi-Buisson N., Fauroux B. (2019). Polysomnographic findings in Rett syndrome. Eur. J. Paediatr. Neurol..

[16] Hagebeuk E.E., Bijlmer R.P., Koelman J.H., Poll-The B.T. (2012). Respiratory disturbances in rett syndrome: Don’t forget to evaluate upper airway obstruction. J. Child Neurol..

[17] Peri F., Cherchi C., Chiarini Testa M.B., Pavone M., Verrillo E., Cutrera R. (2024). The Efficacy of Noninvasive Ventilation in Patients Affected by Rett Syndrome With Hypoventilation. Pediatr. Neurol..

[18] MacKay J., Leonard H., Wong K., Wilson A., Downs J. (2018). Respiratory morbidity in Rett syndrome: An observational study. Dev. Med. Child Neurol..

[19] De Felice C., Guazzi G., Rossi M., Ciccoli L., Signorini C., Leoncini S., Tonni G., Latini G., Valacchi G., Hayek J. (2010). Unrecognized lung disease in classic Rett syndrome: A physiologic and high-resolution CT imaging study. Chest.

